# Epidemiology of midlife fracture: self-reported and hospital episode data from the UK Biobank

**DOI:** 10.1093/jbmr/zjag046

**Published:** 2026-02-27

**Authors:** Catherine Rolls, Helen Dawes, Marcus Munafo, Benjamin G Faber, Emma M Clark

**Affiliations:** Translational and Applied Research Group, University of Bristol, Bristol, BS8 1TU, United Kingdom; Musculoskeletal Research Unit, University of Bristol, Bristol, BS10 5NB, United Kingdom; Department of Physiotherapy, University Hospitals Bristol and Weston NHS Foundation Trust, Bristol, BS2 8HW, United Kingdom; College of Medicine and Health, University of Exeter, Exeter, EX4 4QJ, United Kingdom; Vice Chancellor’s Office, University of Bath, Bath, BA2 7AY, United Kingdom; Musculoskeletal Research Unit, University of Bristol, Bristol, BS10 5NB, United Kingdom; Integrative Epidemiology Unit, University of Bristol, Bristol, BS1 5DS, United Kingdom; Musculoskeletal Research Unit, University of Bristol, Bristol, BS10 5NB, United Kingdom

**Keywords:** adult fracture, epidemiology, UK Biobank, midlife

## Abstract

Fracture risk in midlife (ages 35-65 yr) is underrecognized despite its implications for long-term skeletal health. Using UK Biobank data, we aimed to characterize fracture epidemiology in this critical age group through 2 complementary approaches. We conducted 2 studies: a cross-sectional analysis of self-reported fractures (2006–2010) to estimate annualized incidence risk per 10 000 people and a longitudinal cohort analysis using linked Hospital Episode Statistics (HES; 2001–2022) to calculate incidence rates per 10 000 person-years, both stratified by sex, skeletal site, and 10-yr age bands. Fracture incidence varied substantially by age and sex. Among women, risk increased from age 35 yr and accelerated notably from the mid-40s, peaking at 246 per 10 000 people in the 56–65-yr age group. This female trajectory, emerging earlier than previously recognized, contrasts with men, whose highest fracture risk occurred in early midlife (peak: 232 per 10 000 people; ages 35-45). Across both sexes and age bands, the most reported fracture sites were “other” (including digits and facial bones), wrist, ankle, arm, leg, spine, and hip, in descending order. In HES-linked data, 43 572 fractures were identified. Incidence patterns mirrored those from self-report: higher early midlife fracture rates in men, followed by a transition to female predominance from approximately age 45. This large-scale, dual-method analysis offers the first clear evidence that female fracture risk begins to increase from age 35, with a marked acceleration from 45 yr onward. These sex-specific trajectories in midlife fracture incidence are not fully captured in current clinical models and indicate that further work is needed to determine whether earlier or tailored approaches to risk assessment could provide cost-effective benefit in reducing the burden of fracture in midlife.

## Introduction

Fractures are a growing health concern, with approximately 178 million new fractures per year globally.^1^ Given the considerable mortality and morbidity associated with sustaining a fracture,^2–4^ understanding how the epidemiology of fracture differs by age groups and fracture site is of importance to both health researchers and healthcare commissioners seeking to find new strategies for prevention and treatment. Much of the published data on fracture epidemiology to date has come from research into either children and adolescents or older adults.^5–7^ There is no defined age range for midlife and studies of adults are frequently dichotomized into categories of over or under age 50 yr. We lack granular data on the trajectories of fracture risk during midlife, a crucial stage in relation to changing health status.^8^^,^^9^ Additionally, research on the effect of menopause shows that, for women, midlife represents a pivotal life stage in changes to bone quality,^10^ but little is known about how this affects incident fracture risk.

The UK Biobank study (UKB) is an ongoing longitudinal cohort study in the United Kingdom that recruited 500 000 adults aged 40–69 yr between 2006 and 2010, offering a unique resource for investigating health outcomes in middle-aged adults. The UKB holds extensive self-report data as well as record linkage to hospital admissions data. The richness of these data has resulted in numerous studies with a focus on fractures as either the exposure or outcome of interest: from associations between socioeconomic deprivation, bone health, and fracture^11^ to relationships between fragility fracture and cardiovascular health.^12^ Despite the widespread use of UKB fracture data, to our knowledge no study to date has comprehensively described the epidemiology of fracture within the cohort or assessed how representative these data are in comparison to other published UK fracture epidemiology. Such information is essential for interpreting the context and generalizability of midlife fracture findings derived from the UKB.

This study has 4 aims. First, to describe the epidemiology of fractures and fall-related fractures in adults aged 35–65 yr using self-reported data from the UKB inception cohort (2006–2010). Second, to examine the epidemiology of fractures in the same age group using Hospital Episode Statistics (HES) data from 2001–2023. Third, to assess the validity of self-reported fracture data by comparing self-reported hip fractures with linked HES records during the same period. Finally, to compare the fracture epidemiology in the UKB cohort with findings from other UK-based cohort studies.

## Materials and methods

We preregistered the study protocol with the Open Science Framework.^13^ Changes to the study protocol are described in **Methods S1**. We followed the Strengthening the Reporting of Observational Studies in Epidemiology (STROBE) guidelines^14^ for reporting this study (**Methods S2**).

### Study design and population

Once recruited to the UKB, participants completed both touch-screen questionnaires and verbal interviews and consented to data linkage to hospital records. There are self-reported questionnaire data and linked HES data on just under 500 000 individuals.^15^ For the cross-sectional analysis, we used self-reported fracture data from the UKB baseline assessment, collected between March 13, 2006, and October 1, 2010. For the longitudinal analysis, we used linked inpatient HES data spanning from January 1, 2001, to December 21, 2023. The earlier start date for the longitudinal data was necessary to capture fracture events reported retrospectively in the self-report data (which covered the previous 5 years), enabling a meaningful fracture validation analysis by allowing overlap between the 2 data sources. Ethical approval for the cohort was provided by the National Health Service (NHS) Northwest Multicentre Research Ethics Committee (21/NW/0157). For the cross-sectional analysis of self-reported fracture, we included adults aged 40–65 yr at the baseline visit. We excluded participants who did not answer the question on fractures. For the HES analysis we included all participants who consented to their health data being linked but report on fracture incidence in adults aged 35–65 yr.

For all participants demographic data were collected from the baseline assessment visit and included age at recruitment, sex, ethnicity, study site (country), risk-taking behavior, smoking status, Townsend deprivation index, BMI, and menopausal status. Figure 1 details the number of participants in each part of the study.

**Figure 1:**
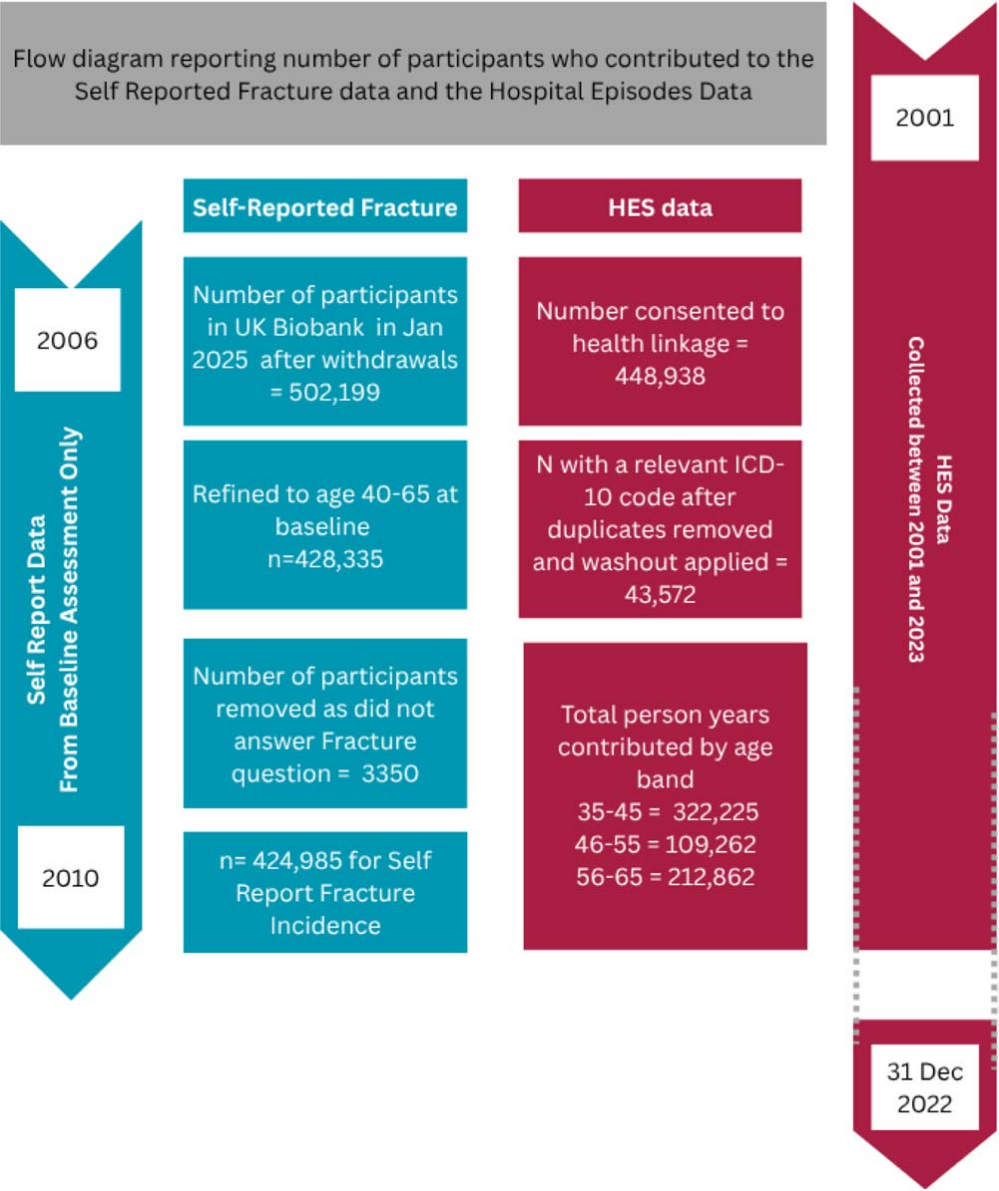
Flow of participants in the study of epidemiology of fracture in the UK Biobank. Abbreviations: HES, Hospital Episode Statistics; ICD-10, *International Classification of Diseases, Tenth Revision*.

### Fracture ascertainment

The self-reported fracture cohort included anyone who had answered yes to the question, “Have you fractured/broken any bones in the last five years?” Two follow-up questions provided information on fracture location (leg, hip, ankle, spine, arm, wrist, other) and whether the fracture was caused by a fall.

Subsequently, we used the inpatient admissions data from the HES database to capture fractures that resulted in an admission to a hospital. The HES database is made up of data from information provided by NHS England’s Admitted Patient Care information for England, the Scottish Morbidity Records for Scotland, and the Patient Episode Database for Wales. All data are provided as *International Classification of Diseases, Tenth Revision* (ICD-10), codes.^16^

Participants were categorized into 10-yr age bands based on their age at the time of fracture. For each self-reported fracture site (as described previously), we compiled a corresponding list of relevant ICD-10 codes. Fractures classified as "other" referred to those occurring at sites not captured by any of the specific self-reported categories; these were predominantly fractures of the hands, feet, skull, and chest. We also created a group of major osteoporotic fractures based on previous literature. To distinguish separate fracture events, we applied a 1-year washout period between fractures assigned the same ICD-10 code. Full details on our approach to fracture ascertainment and classification are detailed in **Methods S3**.

To assess the validity of self-reported fracture data in the UKB, we conducted a validation study focused on hip fractures. We compared self-reported hip fractures with corresponding hospital admissions recorded in the HES dataset, matched by participant over a comparable time period. Hip fracture was chosen because it is a memorable injury and, in the United Kingdom, typically results in hospital admission regardless of age. We considered it the most likely fracture type to be captured in both the self-report and HES datasets.

### Statistical analysis

All data were downloaded and analyzed in R-Studio (version 2025.05.1, Mariposa Orchid). Demographic variables for all UKB participants at baseline assessment were summarized across groupings of fracture or no fracture for male and female sex.

#### Cross-sectional analysis of self-reported fracture data

For each fracture site we calculated the cumulative incidence risk of fracture between 2006 and 2010 in 10-yr age bands—35–45, 46–55, 56–65—based on total number of fractures, and population size was reported separately for male and female sex. The number and percentage of fractures resulting from a simple fall are reported by fracture site and sex. We elected to use complete case analysis for incidence calculations of self-reported fracture, removing participants who did not answer this question. Having looked at the proportion and demographics of nonresponders (Table S1) we determined that the amount of missing data was both likely to be missing at random and minimal and so did not require imputation.

Because the way that the UKB asks participants to identify the site of their fracture does raise the possibility of misclassification, we felt that certain anatomical sites could be confused. To understand this better we conducted a sensitivity analysis (**Methods S4**) between wrist and arm, leg and hip, and leg and ankle fractures.

#### Longitudinal analysis of HES data

For each of the fracture groups described above we calculated the age- and sex-specific incidence rates. Person-years for each age group were calculated for each fracture type separately, from the date the participant entered the study up until they had either sustained a fracture or, if no fracture, until the end date (October 31, 2022). If an individual had multiple episodes of 1 fracture type they were censored at the point of their first fracture of that type. For full details on our statistical approach see **Methods S5**.

For fracture validation we used date-matched HES and self-reported hip fracture data to create Venn diagrams based on occurrence of a hip fracture in both datasets. We then calculated the sensitivity of self-reported hip fracture. R scripts for all of the analyses are available on GitHub (https://github.com/RollsCf/UKB-Fracture-Epi.git).

## Results

### Epidemiology of self-reported fractures by age, sex and fracture site

The demographic characteristics of the baseline cohort of UKB are presented in Table 1. There was a total of 22 963 female and 17 258 male participants aged 35–65 yr who reported a fracture of any type during the 2006 and 2010 baseline assessment, giving a crude annual incidence of 196 per 10 000 people for females and 181 per 10 000 people for males.

**Table 1:** Self-reported fractures (no/yes) in the UK Biobank between 2006 and 2010.

	No Fracture		Fracture		Overall	
	Females (*n* = 211 353; 55%)	Males (*n* = 173 411; 45%)	Females (*n* = 22 963; 57%)	Males (*n* = 17 258; 43%)	Females (*n* = 234 316; 55%)	Males (*n* = 190 669; 45%)
**Age group**						
**35–45 yr**	66 673 (32)	51 827 (30)	5331 (23)	6816 (40)	72 004 (31)	58 643 (31)
**46–55 yr**	89 001 (42)	70 483 (41)	9809 (43)	6571 (38)	98 810 (42)	77 054 (40)
**56–65 yr**	55 679 (26)	51 101 (30)	7823 (34)	3871 (22)	63 502 (27)	54 972 (29)
**Ethnicity**						
**Asian and British Asian**	3160 (2)	3485 (2)	236 (1)	231 (1)	3396 (1)	3716 (2)
**Black and Black British**	3962 (2)	2861 (2)	244 (1)	187 (1)	4206 (2)	3048 (2)
**Mixed**	5379 (3)	3868 (2)	391 (2)	319 (2)	5770 (3)	4187 (2)
**White**	198 265 (94)	162 487 (94)	22 033 (96)	16 447 (95)	220 298 (94)	178 934 (94)
**Location**						
**England**	195 118 (92)	160 604 (93)	21 157 (92)	15 989 (93)	216 275 (92)	176 593 (93)
**Scotland**	15 364 (7)	12 082 (7)	1672 (7)	1178 (7)	17 036 (7)	13 260 (7)
**Wales**	871 (0.4)	725 (0.4)	134 (0.6)	91 (0.5)	1005 (0.4)	816 (0.4)
**Risk**						
**No**	41 195 (20)	58 390 (34)	5522 (24)	7614 (44)	46 717 (20)	66 004 (35)
**Yes**	161 655 (77)	108 922 (63)	16 429 (72)	9011 (52)	178 084 (76)	117 933 (62)
**Smoking**						
**Current**	19 464 (9)	22 117 (13)	2577 (11)	3117 (18)	22 041 (9)	25 234 (13)
**Never**	126 754 (60)	88 097 (51)	12 756 (56)	8214 (48)	139 510 (60)	96 311 (51)
**Previous**	64 430 (31)	62 604 (36)	7539 (33)	5857 (34)	71 969 (31)	68 461 (36)
**Townsend**						
**Mean (SD)**	−1.34 (3.04)	−1.25 (3.15)	−1.12 (3.14)	−0.820 (3.36)	−1.32 (3.05)	−1.21 (3.17)
**BMI, kg/m** ^**2**^						
**Mean (SD)**	27.0 (5.25)	27.9 (4.28)	27.3 (5.34)	27.7 (4.44)	27.0 (5.26)	27.9 (4.30)
**Menopause**						
**Postmenopause**	118 531 (56)	NA	14 798 (64)	NA	133 329 (57)	NA
**Premenopause**	59 003 (28)	NA	4348 (19)	NA	63 351 (27)	NA

Values are No. (%) unless otherwise indicated.

Abbreviation: NA, not available.

When stratified by age and sex there was a clear gradient of increasing fracture incidence throughout midlife in females, with those aged 56–65 yr having the highest fracture incidence of 246.39 per 10 000 per year, whereas the opposite was seen in males, with the highest incidence being in 35–45-yr-old males who have an incidence of 232.46/10 000 per year. At age 46–65 yr, the incidence in females is higher than that of males (Figure 2 and **Results S1**).

**Figure 2:**
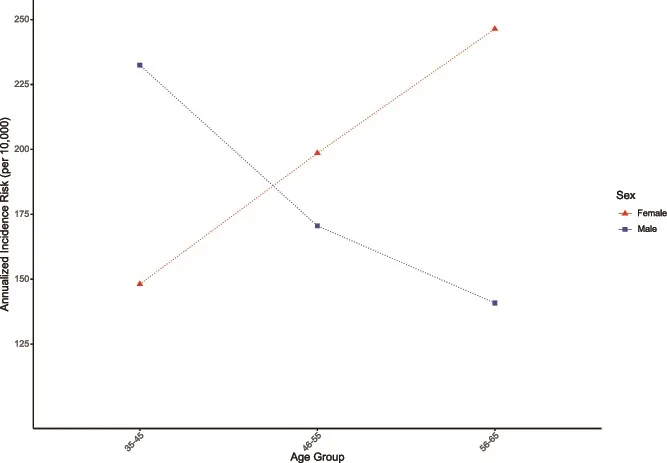
Annualized fracture incidence risk per 10 000 people from self-reported fracture. * note: the dotted line connects the age groups for visual clarity only and does not indicate measured data points between these groups.

The annualized incidence of self-reported fracture between 2006 and 2010 is provided for each anatomical location, by age and sex (Figure 3, **Results S2** and **S3**). While the absolute incidence differs between the sexes by fracture site, the order of most to least common fracture sites is consistent by sex and through the age groups. The highest incidence was recorded for “other” fracture, which includes digit and facial fractures, followed by wrist, ankle, arm, leg, spine, and hip as the least common. We found a strong positive correlation between self-reporting of the following pairs of fractures, arm and wrist, leg and hip, and leg and ankle (**Methods S4**).

**Figure 3:**
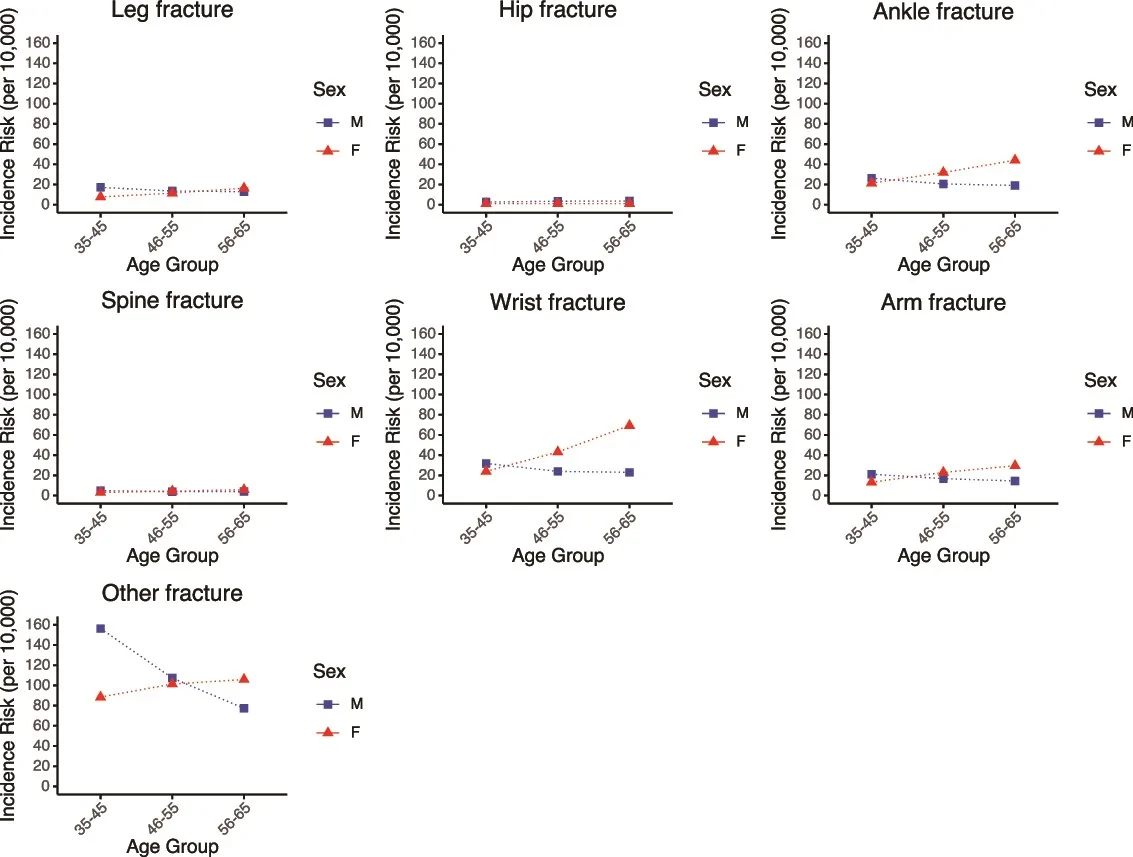
Incidence risk per 10 000 people from self-reported fracture between 2006–2010 reported by sex and age group. The dotted lines connect the age groups for visual clarity only and do not indicate measured data points between these groups.

### Falls

Data on fractures secondary to a fall (**Results S4**) showed that approximately 70% of women, regardless of age, reported their fracture resulted from a fall from standing height or less. Over 70% of female wrist, arm, and ankle fractures at all ages were fall-related. Among men, the proportion of fall-related fractures was lower: 45%–55% of wrist fractures in those aged 35–55 yr were attributed to falls, increasing to 64.5% in the 56–65-yr age group. A similar pattern was observed for male ankle fractures, increasing from 56%–60% in younger age groups to 66% in older men. As we did not analyze all falls data, we are unable to draw conclusions about overall fall risk differences between men and women.

### Epidemiology of fractures from HES 2001–2022

Figure 4 shows the incidence rate per fracture site in the HES dataset from 2001–2022. Full results are available in **Results S5**. As with the self-reported fracture results, the HES data show heterogeneous incidence by fracture site. For all sites, the male incidence is higher in the 35–45-yr age group than in women; this remains the case for all but wrist and ankle fracture in the 46–55-yr age group. In the 56–65-yr age group the trend reverses, with women having the highest incidence of all fractures except for spine and other fracture. The results for a major ostepoporotic fracture in women show a steep escalation from age 45 yr.

**Figure 4:**
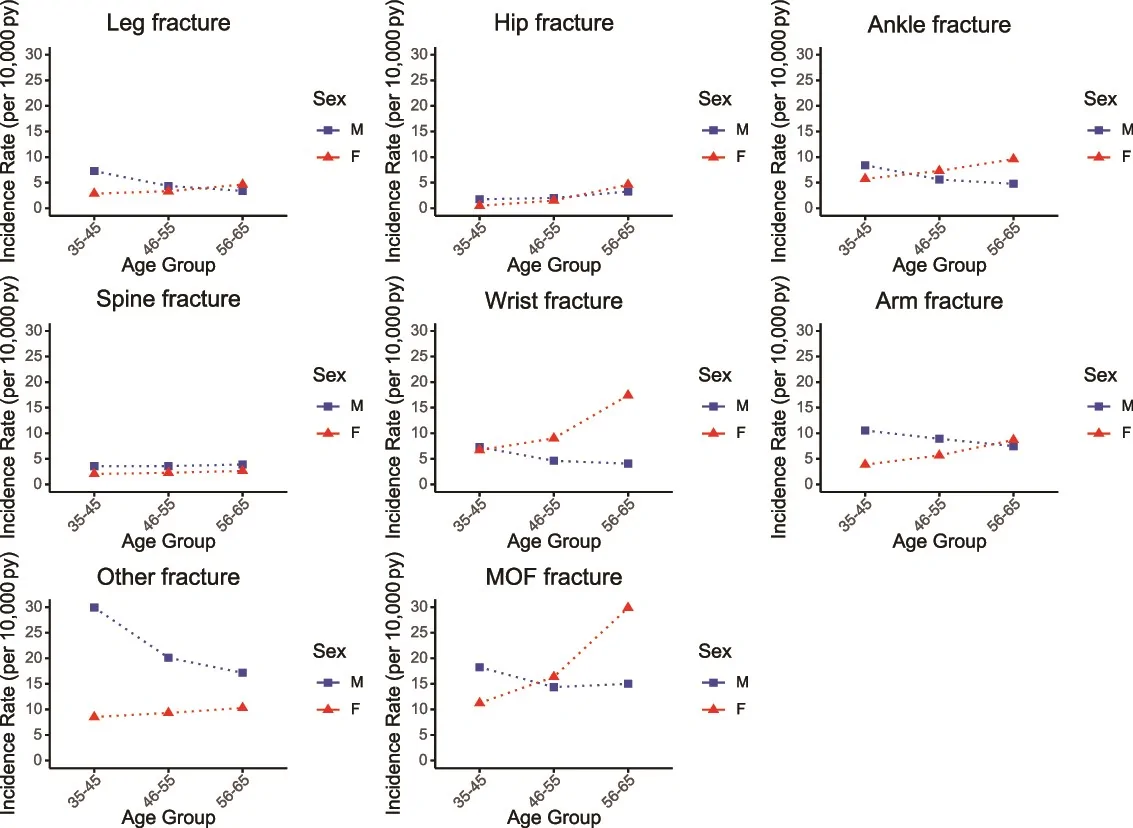
Fracture incidence rate per 10 000 person-years by sex across fracture types based on HES data 2001–2022. The dotted lines connect the age groups for visual clarity only and do not indicate measured data points between these groups. Abbreviations: HES, Hospital Episode Statistics; MOF, major osteoporotic fracture.

The incidence of spine fractures shows the least variation across the age groups in men and women and is lower than all peripheral fractures except for hip. Among women, the HES data align closely with self-reported data, with the ranking of fracture types from most to least common remaining consistent. In contrast, for men, the HES data show some variation, with the order of fracture types differing by age group. Notably, wrist fractures are less common than ankle or arm fractures across all age groups.

### Hip fracture validation

Of 364 hip fractures recorded in HES and 665 self-reported, 287 were found in both datasets (Figure 5). The sensitivity of self-reporting for hip fracture was 0.79, meaning that approximately 80% of actual hip fractures as reported in HES were self-reported. The overreporting proportion, the number of the total population who self-reported a fracture that was not in HES, was 0.09%^.^ This suggests that the effect of false positives on the overall incidence is minimal. We used Venn diagrams to explore reasons to explain the 378 fractures that were not accounted for in the HES dataset (Figure 5). Looking at a broader classification of hip fracture by widening out the ICD-10 criteria to include codes for pelvis and femoral fractures, this reduced the number of hip fractures only captured as self-reported fracture to 300. We also considered whether participants may have reported a hip fracture coded in HES as a leg fracture. This reduced the number of hip fractures only recorded in HES down to 22 (see Table S2 for full details).

**Figure 5:**
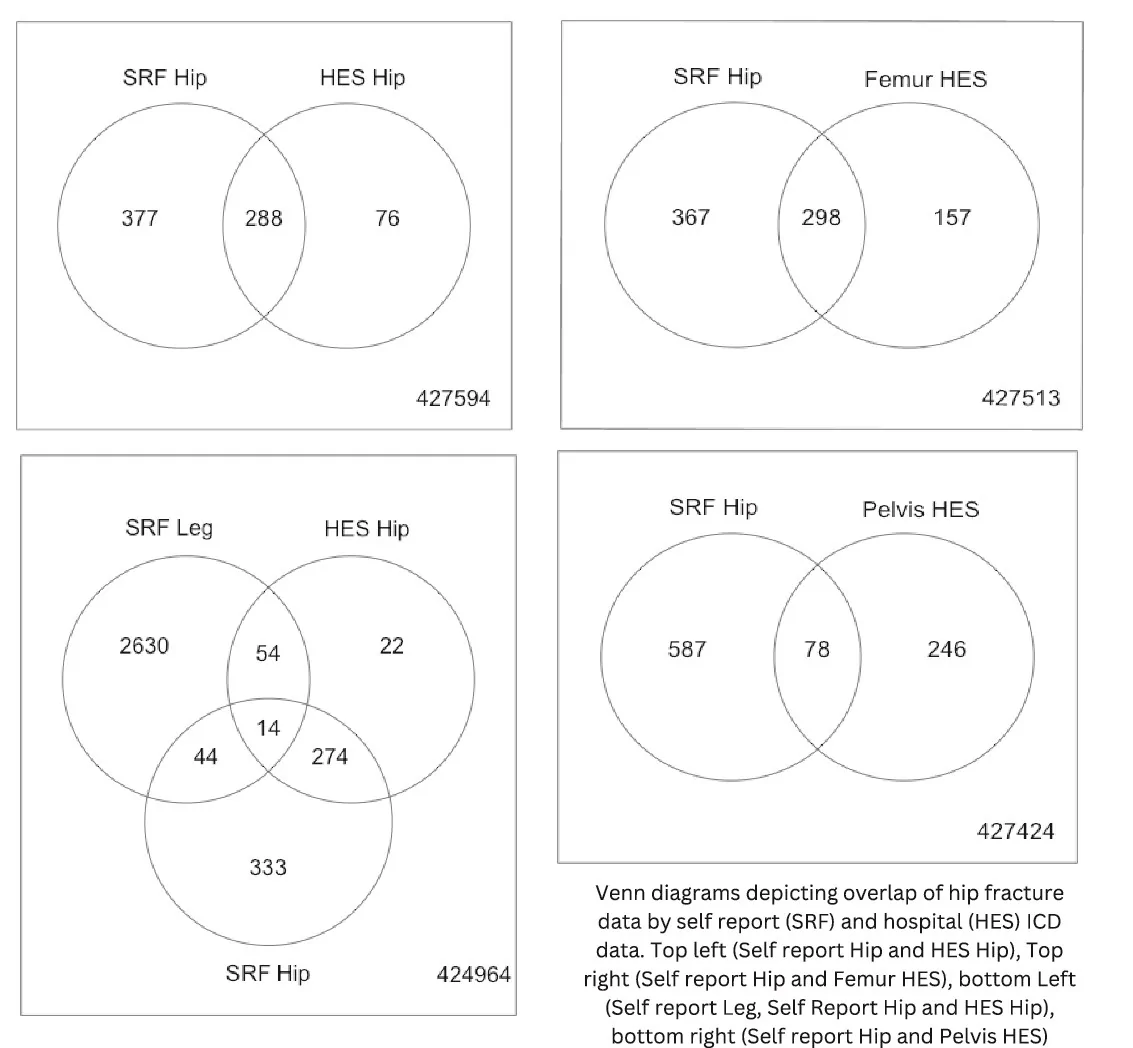
Validation of hip fracture data in the UK Biobank. Abbreviations: HES, Hospital Episode Statistics; ICD, *International Classification of Diseases*.

## Discussion

This study provides the first, to our knowledge, national-level characterization of midlife fracture epidemiology in the United Kingdom using both self-reported and hospital-linked data from the UKB. Most notably, our findings reveal a previously underrecognized trajectory of increasing fracture risk in women beginning as early as age 35 yr, with a marked acceleration from the mid-40s. This female-specific pattern resembles the postmenopausal risk profiles traditionally seen in older populations and suggests that skeletal vulnerability begins earlier than often assumed.

This trend is evident across both data sources and fracture sites, with wrist fractures showing particularly sharp increases in incidence. Given that bone loss associated with menopause can begin during the transition phase, typically starting around age 40 yr,^16^ our findings support the hypothesis that midlife fractures, even at non–weight-bearing sites, may be early clinical markers of menopause-related bone decline.^2^^,^^17^ These insights highlight the need for further research to determine whether sex-specific and potentially earlier approaches to risk assessment could help mitigate long-term skeletal fragility.

In contrast, men demonstrated a different epidemiological pattern: high fracture incidence in early midlife (35–45 yr), particularly for arm and ankle fractures, with a gradual decline thereafter. This trajectory aligns with previous studies attributing early male fracture risk to sports participation and other risk-taking behaviors associated with higher injury risk—for example, driving at excessive speeds.^5^^,^^16^^,^^18–20^ However, our data suggest that elevated risk persists well into midlife, with men maintaining a higher incidence than women until the mid-40s. It is important to acknowledge that the underlying mechanisms driving these observations remain speculative. The limited granularity of data on behavioral contributors to fracture risk in midlife—particularly among women—precludes firm conclusions. As such, we cannot exclude the possibility that risk-taking behaviors contribute to fracture incidence in both middle-aged women as well as men.

When comparing our results from the self-reported fracture data with other studies of annualized incidence risk in the last 40 yr, we found similar fracture trajectories by age and sex, but notably higher absolute incidence rates in the UKB (Figure 6, Table S3).^17^^,^^18^^,^^21^^,^^22^ Several factors may contribute to this difference. First, many earlier UK-based fracture studies were conducted in single geographic regions.^17^^,^^22–24^ Regional studies are subject to local sociodemographic influences, such as age structure, ethnicity, socioeconomic status, and lifestyle, that limit their generalizability. By contrast, UKB provides national-level data, offering a more representative view of fracture epidemiology in the United Kingdom.

**Figure 6:**
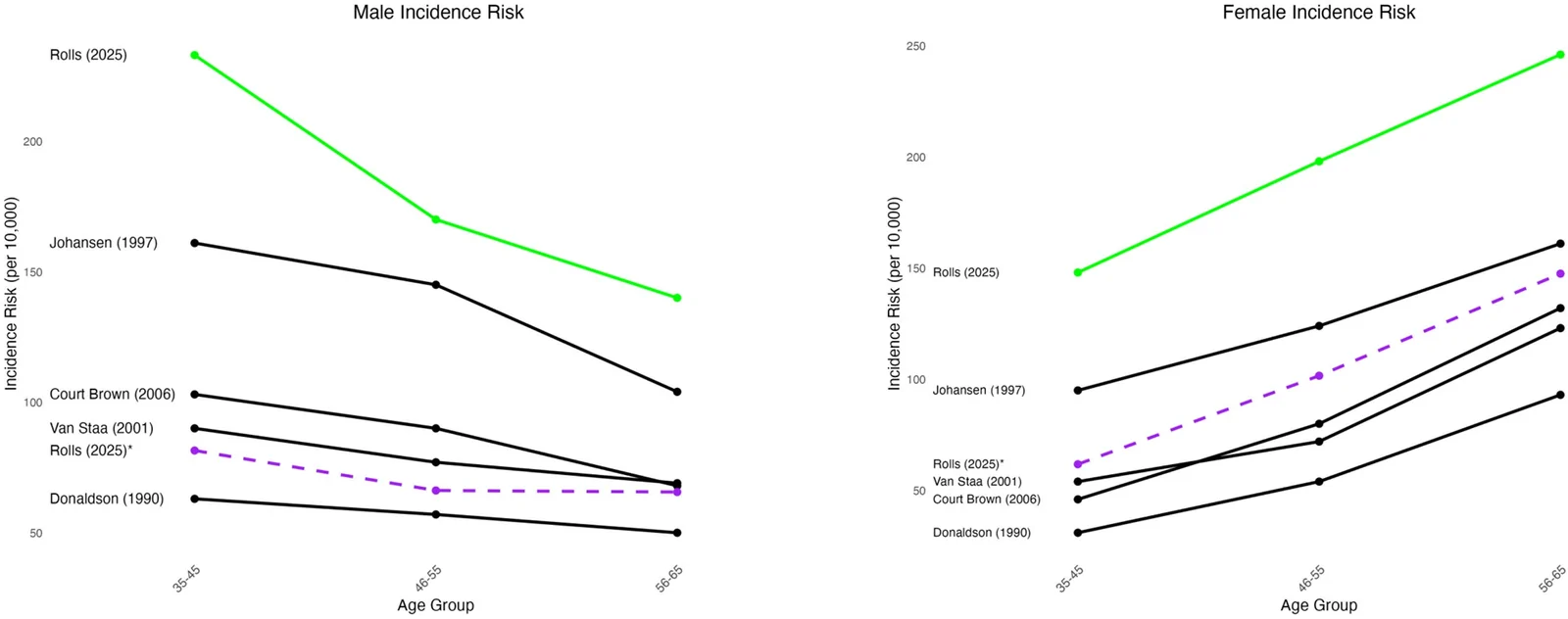
UK studies of fracture risk in the last 40 yr. *The dotted lines represent incidence risk from self-report fracture data in this study when “other” fracture is omitted. See Table S4 for raw data.

The most comparable study to ours in terms of national scope is that of Van Staa and colleagues^21^ who analyzed fracture incidence in the General Practice Research Database (GPRD), now the Clinical Practice Research Database (CPRD), from 1988 to 1998. The CPRD holds primary care data on approximately 6.9% of the population in the United Kingdom. Our fractures incidence is around 2.5 times that of Van Staa and colleagues at every age.^21^ Several explanations may account for this discrepancy. One possibility is the use of self-reported data in UKB, which may lead to overreporting. Our sensitivity analysis showed that anatomically similar fracture pairs were often reported together (eg, a hip and leg fracture), which may reflect double-counting. However, our validation of hip fractures showed reasonable concordance with hospital records, suggesting overall that self-report validity is acceptable, for hip fracture at least. Another explanation is temporal: our study reflects more recent data and may indicate a true increase in fracture incidence over the past 2 decades. Additionally, Van Staa and colleagues excluded fractures to the face, fingers, and toes, sites classified as “other” in UKB. When we recalculated incidence excluding these “other” fractures, the UKB estimates aligned more closely with GPRD data (Figure 6). This adjustment revealed that women’s fracture rates remained elevated, but male rates were lower than initially estimated, underscoring the fracture burden in middle-aged men from skull and digit fractures.

To improve future data quality, we recommend that UKB consider using body charts, a method previously found to increase accuracy of reporting in pain medicine, to reduce potential misclassification or double-reporting of fracture sites.^24^

Our longitudinal analysis using health-linked HES data highlights variability in fracture incidence by anatomical site across midlife. The only other contemporary UK study to also report incidence rate is that of Curtis and colleagues,^7^ who used data from the CPRD between 1988 and 2012 (see Table S4 for full details). While their study stratified adults into broad age groups (18–49 and ≥50 yr), our finer age-band analysis reveals comparable fracture trajectories by site, despite differences in absolute incidence. For example, Curtis and colleagues reported an incidence rate for wrist fracture of 11 per 10 000 person-years in males under 50 yr, whereas we found an incidence rate of 7.29 in those aged 35–45 yr and 4.61 in those aged 46–55 yr. The higher rates observed in their data may reflect the inclusion of younger adults, as adolescent and young-adult males are known to have elevated fracture risk due to sports participation and risk-taking behaviors.^5^^,^^6^ Our narrower age bands show that, although men continue to have relatively high fracture rates in early midlife, risk declines with age, converging with, and eventually being surpassed by, female rates in later midlife.

We also observed that upper limb fracture rates differed more between studies than lower limb fracture rates. This discrepancy may relate to differences in data sources: HES captures inpatient hospital admissions, whereas CPRD includes diagnoses from primary care. Injuries such as upper limb fractures, particularly in younger individuals, may not always result in hospital admission and are therefore likely underrepresented in HES. This highlights the influence of data source on ascertainment and underlines the importance of context when comparing incidence estimates across datasets.

Due to the significant mortality and morbidity associated with hip fracture it is worth highlighting our current estimates of hip fracture incidence. Our data from both HES and self-reported fracture suggest a low incidence of hip fracture for both males and females aged under 65 yr. From our HES data on adults aged 56–65 yr we found there to be an incidence rate (per 10 000 person-years) of 4.6 for females and 3.26 for males. By comparison, Curtis and colleagues reported an incidence rate of 9.9 in males and 28.2 in females over 50 yr. We suggest that the values for the over-50 age group provided by Curtis and colleagues best represent older adults (>65 yr), and that our values are the current best estimates for incidence risk in adults younger than 65 yr. Our validation work on hip fracture further supports the accuracy of these estimates in the UKB dataset. These updated, age-specific estimates from UKB challenge prior assumptions about hip fracture timing and risk in midlife adults and suggest that intervention thresholds currently targeted at older adults may miss an emerging at-risk population in their late 50s to early 60s.

### Limitations

There are known limitations with using the UKB, in particular its lack of representativeness, with the majority of participants being of White ethnicity and tending to be more affluent and healthier than the general population,^25^ making it less representative of the wider UK public. We also acknowledge that the HES data are only capturing admissions episodes, which most likely contributes to the inconsistencies in peripheral fracture sites seen here. In this younger cohort it is likely that fractures such as wrist, arm, and ankle would be managed on an outpatient basis and would not be captured in HES. We also lacked information on premature menopause, ovarian failure, or secondary osteoporosis as potential causes of increased fracture risk, an area that would be interesting to explore further.

One of our aims was to investigate the epidemiology of fall-related fractures in middle-aged adults. A limitation of this study is the restricted information on fracture mechanism in the UKB. While the dataset includes self-reported falls as a cause of fracture, it lacks detail on other injury mechanisms, limiting our ability to interpret causality. Our findings suggest that women report more fall-related fractures than men at all ages, but this may reflect sex differences in reporting rather than true differences in injury cause. A more nuanced understanding of fracture epidemiology in midlife requires more comprehensive and standardized recording of injury mechanisms in large-cohort studies.

## Conclusion

This study provides new insight into midlife fracture trajectories, revealing distinct, sex-specific patterns. For the first time, to our knowledge, we show that fracture risk in women increases from age 35 yr, with a sharp acceleration from the mid-40s, trajectories that are not fully reflected in current clinical models or guidelines. In contrast, men retain the higher fracture risk characteristic of younger males into their fifth decade, particularly for upper and lower limb fractures, before experiencing a gradual decline. These findings challenge conventional assumptions about the timing of skeletal vulnerability and highlight the need for earlier, sex-specific approaches to fracture prevention. It is important to note that we use the term “skeletal vulnerability” descriptively rather than diagnostically, as we wish to represent the higher-than-expected fracture incidence. The pattern does, however, prompt further questions as to how these fractures may relate to osteoporosis. Our study did not include measures of bone mineral density or bone architecture; as such, this question falls beyond the scope of the current analysis but is a clear priority for future research.

By leveraging both self-reported and hospital-linked data from the UKB, we demonstrate the complementary value of combined data sources in musculoskeletal epidemiology. While HES may under-ascertain fractures not requiring hospital admission, our validation analyses suggest that self-reported fracture data are sufficiently reliable for population-level estimation.

Our age- and site-specific incidence estimates, reported in detailed 10-yr bands, provide a contemporary and granular picture of midlife fracture risk in the United Kingdom. These findings have important implications for clinical risk assessment, healthcare resource planning, and the design of future interventions aimed at reducing fracture burden in adults.

## Supplementary Material

Supplementary_info_zjag046

## Data Availability

Full data relating to this article are available within the supplementary information files.

## Code availability

Code used for analysis described in this manuscript is available at https://github.com/RollsCf/UKB-Fracture-Epi.git.
