# Supplementary material for: Epidemiology of midlife fracture: self-reported and hospital episode data from the UK Biobank
Source: J Bone Miner Res. 2026 Feb 27;41(11):1209–17. doi: 10.1093/jbmr/zjag046 (PMC13716911; doi:10.1093/jbmr/zjag046)
Supplement: Supplementary file 1 [file supplementary_info_zjag046.pdf]

Epidemiology of Fracture in Middle Aged Adults: Self-Reported and Hospital Episode Data from the UK Biobank (2006-2023).

Catherine Rolls[1,2,3], Helen Dawes[4], Marcus Munafo[5], Benjamin G Faber[2,5], Emma M Clark[2]

1. Translational and Applied Research Group, University of Bristol
2. Musculoskeletal Research Unit, University of Bristol
3. University Hospitals Bristol and Weston NHS Foundation Trust
4. University of Exeter
5. Vice Chancellor's Office, University of Bath
6. Integrative Epidemiology Unit, University of Bristol

Supplementary info

Methods

eMethods 1 Documentation of changes to the study protocol

eMethods 2 The Strengthening of Reporting of Observational Studies in Epidemiology (STROBE) checklist

eMethods 3 Rational for fracture ascertainment and classification

eMethods 4: Sensitivity analysis (misclassification)

eMethods 5. Calculations of Incidence risk and rate

Tables

eTable 1: Missing fracture data by demographics

eTable 2 Sensitivity and specificity analysis of HESIN vs SRF for hip fracture

eTable 3: Annual incidence per 10,000 people from UK studies

eTable4: Site specific fracture incidence rate per 10,000 person years by age and sex from Curtis and colleagues (CPRD) and HESIN data from UKBB

Results

eResults 1 Annualized Fracture Incidence Risk per 10,000 People from self-reported fracture

eResults 2 Female Incidence Risk per 10,000 people from Self-Reported fracture between 2006-2010 reported by age group.

eResults 3 Male Incidence Risk per 10,000 people from Self-Reported fracture between 2006-2010 reported by age group.

eResults4: Number (Percentage) of Self-Reported Fractures from Fall - UKBB 2001-2006 by age and sex

eResults 5 Fracture Incidence rate per 10,000 person years by sex across fracture types based on HES data 2001-2022

eMethods 1. Documentation of changes to the study protocol

We have followed guidance provided on reporting and justifying deviations from the study protocol.(Moreau and Gamble, 2022)

Deviation (1) Study Aims: Increase the upper age for epidemiology to 65

Description	All incidence calculations have been done for adults aged 35-65 instead of 35-60.
Justification	Changing the upper bound has allowed us to calculate categories of 10-year incidence risk/rate which are comparable to other reported UK data.
Impact	**Low**: We do not feel this has changed the key findings of this study.

Deviation (2) Study Aims: We have compared data to UK studies only

Description	Aim 4 was to compare our result to existing UK and Global studies, we have limited this to comparison of UK studies only
Justification	Due to our understanding of the UK healthcare system we have been able to make informed analysis as to why study results may have differed to ours, particularly in relation to methods of data collection. We particularly wanted to establish how representative the UKBB is of UK fracture epidemiology and felt that comparing to global studies would not add to this.
Impact	**Moderate**: Our findings allow understanding of the UKBB data in the context of UK fracture epidemiology but limit our knowledge of how representative these figures are on a global scale.

Deviation (3) Confounding Variables: Change to confounding variables collected

Description	- Dropped Charlson comorbidity Index<br>- Added Alcohol<br>- Added Smoking
Justification	1) Calculation of the CCI involves detailed analysis of all HES data per participant. Due to time restrictions, we were unable to do this piece of work for this study. It did not form part of the analysis but was to add more context to the demographics table.<br>2) Addition of alcohol and smoking felt appropriate for a middle-aged cohort analysis
Impact	**Low:** This did not affect our calculations of fracture incidence or interpretation of findings. Previous published research does highlight that the UKB is a 'healthy cohort' and we have included this in our discussion of limitations.

Deviation (4) Descriptive statistics for self-reported fracture

Description	The exposure time during the 5 years will be calculated for each age cohort as 5 times number of participants in each age bracket at the baseline assessment centre – we changed this to reporting an annualised incidence risk.
Justification	We felt that a cross-sectional analysis was the best approach to handling this data. Rather than multiplying the number of people in the cohort x 5 for each year in the cohort, we divided the total population by 5 to calculate an annualised incidence risk.
Impact	**Moderate:** This gives us an annualised incidence risk, rather than an incidence risk over 5 years. We felt this was easier to comprehend and was comparable to similar research in the field.

Deviation (5) Reported incidence in 10 years not 5-year age bands

Description	Our analysis plan was to look at incidence in 5-year age bands, we changed this to 10 years.
Justification	We felt on balance that 10-year age bands allow us to highlight important changes with age in mid-life but keep number of fractures per age group and fracture site to

	be of a large enough number to be meaningful. We did not feel that 5-year age bands would add to this and would have been harder to compare with other published data.
Impact	**Low**: We did not feel this impacted on the interpretation of our findings.

eMethods 2. The STROBE checklist

checklist of items that should be included in reports of observational studies

	Item No.	Recommendation	Page No.
**Title and abstract**	1	(*a*) Indicate the study's design with a commonly used term in the title or the abstract	Title page
		(*b*) Provide in the abstract an informative and balanced summary of what was done and what was found	Abstract
**Introduction**			
Background/rationale	2	Explain the scientific background and rationale for the investigation being reported	Introduction
Objectives	3	State specific objectives, including any prespecified hypotheses	Introduction
**Methods**			
Study design	4	Present key elements of study design early in the paper	Methods
Setting	5	Describe the setting, locations, and relevant dates, including periods of recruitment, exposure, follow-up, and data collection	Study design and population
Participants	6	(*a*) *Cohort study*—Give the eligibility criteria, and the sources and methods of selection of participants. Describe methods of follow-up<br>*Case-control study*—Give the eligibility criteria, and the sources and methods of case ascertainment and control selection. Give the rationale for the choice of cases and controls<br>*Cross-sectional study*—Give the eligibility criteria, and the sources and methods of selection of participants	Study design and population, fracture ascertainment
		(*b*) *Cohort study*—For matched studies, give matching criteria and number of exposed and unexposed<br>*Case-control study*—For matched studies, give matching criteria and the number of controls per case	NA
Variables	7	Clearly define all outcomes, exposures, predictors, potential confounders, and effect modifiers. Give diagnostic criteria, if applicable	Fracture ascertainment and eMethods 3
Data sources/ measurement	8*	For each variable of interest, give sources of data and details of methods of assessment (measurement). Describe comparability of assessment methods if there is more than one group	*Statistical analysis including validation description*
Bias	9	Describe any efforts to address potential sources of bias	eMethods 4 and eMethods 5
Study size	10	Explain how the study size was arrived at	Figure 1
Quantitative variables	11	Explain how quantitative variables were handled in the analyses. If applicable, describe which groupings were chosen and why	eMethods 3

Statistical methods	12	(*a*) Describe all statistical methods, including those used to control for confounding	Stat analysis, eMethods 3 and eMethods 4
		(*b*) Describe any methods used to examine subgroups and interactions	Stat analysis plan for reporting by sex and site
		(*c*) Explain how missing data were addressed	Stat analysis and eTable 1
		(*d*) *Cohort study*—If applicable, explain how loss to follow-up was addressed<br>*Case-control study*—If applicable, explain how matching of cases and controls was addressed<br>*Cross-sectional study*—If applicable, describe analytical methods taking account of sampling strategy	NA
		(*e*) Describe any sensitivity analyses	eMethods 4, eTable 3
		(*d*) *Cohort study*—If applicable, explain how loss to follow-up was addressed<br>*Case-control study*—If applicable, explain how matching of cases and controls was addressed<br>*Cross-sectional study*—If applicable, describe analytical methods taking account of sampling strategy	NA
Participants	13*	(a) Report numbers of individuals at each stage of study—eg numbers potentially eligible, examined for eligibility, confirmed eligible, included in the study, completing follow-up, and analysed	Figure 1
		(b) Give reasons for non-participation at each stage	Figure 1
		(c) Consider use of a flow diagram	As above
Descriptive data	14*	(a) Give characteristics of study participants (eg demographic, clinical, social) and information on exposures and potential confounders	Table 1
		(b) Indicate number of participants with missing data for each variable of interest	eTable 1
		(c) *Cohort study*—Summarise follow-up time (eg, average and total amount)	eResults 5 - Calculation of person years per fracture site for HES data
Outcome data	15*	*Cohort study*—Report numbers of outcome events or summary measures over time	eResults *1-5*
		Case-control study—Report numbers in each exposure category, or summary measures of exposure	
		Cross-sectional study—Report numbers of outcome events or summary measures	
Main results	16	(*a*) Give unadjusted estimates and, if applicable, confounder-adjusted estimates and their precision (eg, 95% confidence interval). Make clear which confounders were adjusted for and why they were included	NA
		(*b*) Report category boundaries when continuous variables were categorized	NA
		(*c*) If relevant, consider translating estimates of relative risk into absolute risk for a meaningful time period	NA

Main results	16	(*a*) Give unadjusted estimates and, if applicable, confounder-adjusted estimates and their precision (eg, 95% confidence interval). Make clear which confounders were adjusted for and why they were included	NA
		(*b*) Report category boundaries when continuous variables were categorized	NA
		(*c*) If relevant, consider translating estimates of relative risk into absolute risk for a meaningful time period	NA
Other analyses	17	Report other analyses done—eg analyses of subgroups and interactions, and sensitivity analyses	eTable 3 - HES and SRF sensitivity analysis
**Discussion**			
Key results	18	Summarise key results with reference to study objectives	Discussion
Limitations	19	Discuss limitations of the study, taking into account sources of potential bias or imprecision. Discuss both direction and magnitude of any potential bias	Limitations
Interpretation	20	Give a cautious overall interpretation of results considering objectives, limitations, multiplicity of analyses, results from similar studies, and other relevant evidence	Discussion and conclusion
Generalisability	21	Discuss the generalisability (external validity) of the study results	Discussion<br>Limitations - Reference to other UK studies/limitations of UKB
**Other Information**			
Funding	22	Give the source of funding and the role of the funders for the present study and, if applicable, for the original study on which the present article is based	Declarations and acknowledgements

eMethods 3: Rational for fracture ascertainment and classification

Self-Reported Fracture

We used data from adults aged 40-65 at Assessment centre 0.
Self-reported fracture questions were asked retrospectively. Participants were asked about fractures in the last 5 years. Age groups for self-reported fracture were therefore calculated as age at the assessment centre visit minus 5 years, grouped into 10-year age bands. This would give data on fractures from ages 35-65.

For example, someone aged 65 at the assessment centre would end up in the 56-65 age group, while someone aged 40 at the assessment centre would end up in the 35-45 age group as we felt this best represented the age they were at the time of fracture.

Participants were asked to select the fracture site(s) from a drop-down menu with 7 locations provided (Hip, Leg, Ankle, Wrist, Arm, Spine, Other).
They were not able to select the same site more than once, for example if they had sustained two wrist fractures during that time only one would be recorded, but if they had sustained a wrist and hip fracture both sites could be selected. The second follow up question asked, *'did the fracture result from a simple fall (i.e. from standing height)?'*

Hospital Episode Statistics (HES)

For each participant there may be multiple events, for each event we used the episode start date as the proxy for injury date, when this was not present we used the admissions date instead. As patients can be re-admitted for the same injury some injuries are captured in the HES dataset twice. To avoid this, we used a one-year washout period between fractures with the same ICD-10 code.
As HES does not distinguish between right and left sides, bilateral fractures were treated as one fracture.

Age at injury was calculated from year and month of birth and date of injury as described above. Participants were placed into 10 year age bands based on their age at injury.

For details of ICD-10 codes used to create anatomical categories see the pre-registered study protocol.(Rolls, C, Clark, E and Munafo, M. R., 2024) We have defined Major Osteoporotic Fracture (MOF) as a fracture of the proximal humerus, wrist, spine (not C-Spine), pelvis and distal leg (not ankle) due to their relationship with osteoporotic BMD. (Morin, Lix and Leslie, 2014) While there is some evidence that other fracture sites such as clavicle and ribs can be related to low BMD these findings are inconsistent between studies so we have not included them.

Table A1. Self-reported fracture site mapped to ICD-10 codes. (Full details are available in our pre-registered analysis plan)(Rolls, C, Clark, E and Munafo, M. R., 2024)

Self-Report	Mapped to ICD-10	
Ankle	S82.3	Fracture of lower end of tibia
	S82.5	Fracture of medial malleolus
	S82.6	Fracture of lateral malleolus
	S82.8	Fractures of other parts of lower leg
Leg	S72.3	Fracture of shaft of femur
	S72.4	Fracture of lower end of femur
	S72.7	Multiple fractures of femur
	S72.8	Fractures of other part of femur
	S72.9	Fracture of femur, part unspecified
	S82.0	Fracture of patella
	S82.1	Fracture of upper end of tibia
	S82.2	Fracture of shaft of tibia
	S82.4	Fracture of fibula alone

	S82.7	Multiple fractures of lower leg
	S82.9	Fractures of lower leg, part unspecified
Hip	S72.0	Fracture of neck of femur
	S72.1	Pertrochanteric fracture
	S72.2	Subtrochanteric fracture
Spine	S12.0	Fracture of first cervical vertebra
	S12.1	Fracture of second cervical vertebra
	S12.2	Fracture of other specified cervical vertebra
	S12.7	Multiple fractures of cervical spine
	S12.8	Fracture of other parts of neck
	S12.9	Fracture of neck, part unspecified
	S22.0	Fracture of thoracic vertebra
	S22.1	Multiple fractures of thoracic spine
	S32.0	Fracture of lumbar vertebra
	S32.1	Fracture of sacrum
	S32.2	Fracture of coccyx
	S32.3	Fracture of ilium
	S32.7	Multiple fractures of lumbar spine and pelvis
	S32.8	Fracture of other/unspecified parts of lumbar spine and pelvis
Wrist	S52.5	Fracture of lower end of radius
	S52.6	Fracture of lower end of both radius and ulna
	S62.0	Fracture of scaphoid
Arm	S42.0	Fracture of the clavicle
	S42.1	Fracture of the scapula
	S42.2	Fracture of the upper end of the humerus
	S42.3	Fracture shaft of humerus
	S42.4	Fracture lower end of humerus
	S42.7	Multiple fractures of clavicle, scap and humerus
	S42.8	Fracture of other parts of shoulder and upper arm
	S42.9	Fracture of shoulder girdle part unspecified
	S52.0	Fracture of upper end of ulnar
	S52.1	Fracture of upper end of radius
	S52.2	Fracture of shaft of ulna
	S52.3	Fracture of shaft of radius
	S52.4	Fracture of shafts of both ulna and radius
	S52.7	Multiple fractures of forearm
	S52.8	Fracture of other parts of forearm
	S52.9	Fracture of forearm - part unspecified
	S52.7	Multiple fractures of forearm
Other	S02.0	Fracture of vault of skull
	S02.1	Fracture of base of skull
	S02.2	Fracture of nasal bones
	S02.3	Fracture of orbital floor
	S02.4	Fracture of malar and maxillary bones
	S02.6	Fracture of mandible
	S02.7	Multiple fractures involving skull and facial bones
	S02.8	Fractures of other skull and facial bones
	S02.9	Fracture of skull and facial bones, part unspecified
	S22.2	Fracture of sternum
	S22.3	Fracture of rib
	S22.4	Multiple fractures of ribs
	S22.5	Flail chest
	S22.8	Fracture of other parts of bony thorax
	S22.9	Fracture of bony thorax, part unspecified
	S32.4	Fracture of acetabulum
	S32.5	Fracture of pubis
	S62.2	Fracture of first metacarpal
	S62.3	Fracture of other metacarpal

	S62.4	Multiple fractures of metacarpal bones
	S62.5	Fracture of the thumb
	S62.6	Fracture of other fingers
	S62.7	Multiple fractures of fingers
	S62.8	Fracture other unspecified part of hand and wrist
	S92.0	Fracture of calcaneus
	s92.1	Fracture of talus
	S92.2	Fracture of other tarsal bone
	S92.3	Fracture of metatarsal bone
	S92.4	Fracture of great toe
	S92.5	Fracture of other toe
	S92.7	Multiple fractures of foot
	S92.9	Fracture of foot, unspecified
	S62.1	Fracture of other carpal bone(s)

Table A2. Major Osteoporotic Fracture as based on FRAX (Prox humerus, Wrist, Hip, Vertebra) mapped to ICD-10 codes.

Region	ICD-10 Code	Description
Proximal Humerus	S42.2	Fracture of the upper end of the humerus
	S42.3	Fracture shaft of humerus
Wrist	S52.2	Fracture of shaft of ulna
	S52.3	Fracture of shaft of radius
	S52.4	Fracture of shafts of both ulna and radius
	S52.5	Fracture of lower end of radius
	S52.6	Fracture of lower end of both radius and ulna
Hip	S72.0	Fracture of neck of femur
	S72.1	Pertrochanteric fracture
	S72.2	Subtrochanteric fracture
Vertebra	S22.0	Fracture of thoracic vertebra
	S22.1	Multiple fractures of thoracic spine
	S32.0	Fracture of lumbar vertebra
	S32.7	Multiple fractures of lumbar spine and pelvis

Table A3. Hip fracture self-report mapped to ICD-10 codes.

Region	ICD-10 Code	Description
Hip	S72.0	Fracture of neck of femur
	S72.1	Pertrochanteric fracture
	S72.2	Subtrochanteric fracture
	S72.3	Fracture of shaft of femur

eMethods 4. Calculations of Incidence risk and rate

Self-Reported Fracture (SRF) Analysis

A cross-sectional analysis of Self-Reported Fracture (SRF) data was conducted for each fracture site to estimate the cumulative incidence risk between 2006 and 2010. Analyses were stratified by 10-year age bands (35–45, 46–55, 56–65 years) and reported separately for males and females. The cumulative incidence was calculated based on the total number of reported fractures and the population size within each group. In addition, the number and percentage of fractures attributed to a simple fall were reported by fracture site and sex.

To estimate the annualised incidence risk, the 5-year cumulative incidence was divided by 5, resulting in the following expression:

Annualised incidence risk per 10,000 people = (Number of fractures / Population) × 2,000

Longitudinal Analysis of HES data: Incidence Rate per 10,000 Person-Years

Incidence rates were calculated by dividing the number of individuals with a fracture within each 10-year age band by the total person-years contributed by individuals in that band. Person-years were calculated based on the time each participant spent in a given age band, defined by their age at the start (i.e., age at assessment center) and their age at the end of follow-up. The end of follow-up was determined either by the cohort end date or, if a fracture occurred, the date of the fracture.

Person-year calculations were performed separately for each fracture site of interest. The incidence rate per 10,000 person-years was calculated as:

Incidence rate per 10,000 person-years = (Number of fractures / Total person-years) × 10,000

Full R code is available at https://github.com/RollsCf/UKB-Fracture-Epi.git

eMethods 5: Sensitivity analysis (misclassification)

We considered that there might be misclassification of self-reported fracture with overlap between the following fracture groups

1) Ankle and Leg
2) Hip and Leg
3) Wrist and Arm

Odds Ratio Summary Table

Fracture Pair	Odds Ratio	95% CI Lower	95% CI Upper
Ankle_Leg	15.38	13.86	17.08
Hip_Leg	15.13	11.53	19.86
Wrist_Arm	8.04	7.31	8.85

eTable 1: Missing fracture data by demographics

Complete case analysis is considered acceptable when missing data is less than 5% and can plausibly be treated as missing at random.(Bennett, 2001) Here we follow the reporting guidelines as suggested by Sterne and colleagues (Sterne *et al.*, 2009) and demonstrate that the percentage of missing data across outcomes of interest is small, as well as the distribution of missing data by other demographic variables (see eTable1).

Outcome variable	Total	Number of NA	Percentage of NA	Imputation needed
Falls	428,335	2,321	0.54	No
Fracture Yes/No	428,335	2,566	0.56	No

There was no NA data for fracture site, everyone who reported a fracture completed this question.

	Missing Fracture data stratified by age and confounders					
N =	**Female (NA)**	**Female (Total)**	**Percentage NA**	**Male (NA)**	**Male (Total)**	**Percentage NA**
	1,558	235,874	< 1%	1792	192,461	1%
**Age Group**						
35-45	583	72,587	< 1%	785	59,428	1%
46-55	627	99,437	< 1%	643	77,697	1%
56-65	348	63,850	1%	364	55,336	1%
**Ethnicity**						
Asian and British Asian	56	3,452	2%	61	3,777	2%
Black and Black British	55	4,261	1%	54	3,102	2%
Mixed	75	5,845	1%	68	4,255	2%
White	919	221,217	< 1%	1153	180,087	1%
**Location**						
England	1,470	217,745	1%	1674	178,267	1%
Scotland	81	17,117	< 1%	100	13,360	1%
Wales	7	1,012	1%	18	834	2%
**Risk**						

No	240	46,957	1%	519	66,523	1%
Yes	696	178,780	< 1%	647	118,580	1%
**Smoking**						
Current	159	22,200	1%	290	25,524	1%
Never	638	140,148	< 1%	625	96,936	1%
Previous	316	72,285	< 1%	437	68,898	1%
**Townsend**						
Mean (SD)	0.269 (3.65)	-1.31 (3.06)		0.295 (3.71)	-1.19 (3.18)	
**BMI**						
Mean (SD)	28.2 (6.09)	27.0 (5.26)		27.9 (4.72)	27.9 (4.30)	
**Menopause**						
Do not know	740	38,376	1%	Not applicable	Not applicable	Not applicable
Post menopause	544	133,873	< 1%	Not applicable	Not applicable	Not applicable
Pre menopause	274	63,625	< 1%	Not applicable	Not applicable	Not applicable

eTable 2 Sensitivity and specificity analysis of HESIN vs SRF for hip fracture

	True Positive (TP)	False Positive (FP)	True Negative (TN)	False Negative (FN)	False Positive Rate	False Negative Rate	Sensitivity	Specificity
Just HES Hip and SRF	336	329	6353	380	0.495 (50%)	0.53 (53%)	0.47 (0.43-0.50)	0.95 (0.94-0.95)
Full set accounting for misdiagnosis/reporting	441	225	6165	380	0.338 (34%)	0.46 (46%)	0.54 (0.50-0.57)	0.96 (0.96-0.97)

eTable 3: Annual incidence per 10,000 people (Table adapted from Van Staa 2001)

Author/date	Study years	Study Duration	Location	Fractures recorded	Fracture ascertain ment	35-45		46-54		55-64	
						Male	Female	Male	Female	Male	Female
**Donaldson (1990)**	1980-1992	12 years	Leicester	All fractures	Radiology	63	31	57	54	50	93
**Van Staa (2001) (person years) – No fingers/face**	1988-1998	10 years	GPRD	No finger of facial fractures	Data linkage	90	54	77	72	69	123
**Court Brown (2006)**	2000	1 year	Edinburgh	All fractures	Radiology	103	46	90	80	68	132
**Johansen (1997)***	1994	1 year	Cardiff	All fractures	Radiology	161	95	145	124	104	161
**Rolls (25)**	2006-2010	5 years	UKBB	Arm, Wrist, Spine, Ankle, Leg, Hip, Other	Self-report (all)	233	148	170	198	140	246
**Rolls (25) Minus (‘other fracture’)**	2006-2010	5 years	UKBB	Arm, Wrist, Spine, Ankle, Leg, Hip	Self-report	81.5	61.8	66.2	101.6	65.6	147.5

**denotes where incidence has been estimated from graphical results in paper.*

eTable4: Site specific fracture incidence rate per 10,000 person years by age and sex from Curtis and colleagues (CPRD) and HESIN data from UKBB

		**Age**	**Any**	**Hip**	**Wrist**	**Proximal Humerus**	**Ankle**	**MOF**
**Male incidence per 10,0000 person years**	Curtis (CPRD)	18-49	94.8	0.6	11	6.2	7.4	24.3
	Rolls HESIN (UKBB)	35-45		1.74	7.29	2.01	8.41	
	Rolls HESIN (UKBB)	46-55		2.01	4.61	2.01	5.61	
	Curtis (CPRD)	Over 50	71.8	9.9	8.9	7.9	4.5	38.4
	Rolls HESIN (UKBB)	56-65		3.26	4.06	2.19	4.79	
	Rolls HESIN (UKBB)	Over 65		9.32	3.34	2.82	3.64	
**Female incidence per 10,0000 person years**	Curtis (CPRD)	18-49	54.2	0.3	9.1	4.4	3.5	17.2
	Rolls HESIN (UKBB)	35-45		0.47	6.68	0.72	5.74	
	Rolls HESIN (UKBB)	46-55		1.49	9.01	1.76	7.31	
	Curtis(CPRD)	Over 50	155.4	28.2	39.7	21.7	8.3	98.6
	Rolls HESIN (UKBB)	56-65		4.60	17.41	3.53	9.61	
	Rolls HESIN (UKBB)	Over 65		17.46	20.92	7.08	9.82	

eResults 1: **Annualized Fracture Incidence Risk per 10000 People**

Sex	Age Group	Total Population	Fracture Cases	5-Year Incidence Risk (per 10000)	Annualized Incidence Risk (per 10000)
Female	35-45	72587	5331	734.43	146.89
Female	46-55	99437	9809	986.45	197.29
Female	56-65	63850	7823	1225.22	245.04
Male	35-45	59428	6816	1146.93	229.39
Male	46-55	77697	6571	845.72	169.14
Male	56-65	55336	3871	699.54	139.91

eResults 2: Annualised Fracture Incidence per 10,000 people (Female) from self-reported fracture

**Age**	**Age 35-45 (n= 72004)**		**Age 46-55 (n= 98810)**		**Age 56-65 (n= 63502)**		**Total Female (n=234,316)**	
Fracture Type	Cases	Risk (%)	Cases	Risk (%)	Cases	Risk (%)	Total Cases	Total Risk (%)
Leg	277	8	566	11	519	16	1,362	12
Hip	40	1	140	3	177	6	357	3
Ankle	771	21	1,573	32	1,400	44	3,744	32
Spine	110	3	216	4	183	6	509	4
Wrist	863	24	2,130	43	2,200	69	5,193	44
Arm	477	13	1,131	23	938	30	2,546	22
Other	3184	88	5,008	101	3,361	106	11,553	99

eResults 3: Annualised Fracture Incidence per 10,000 people (Male) from self-reported fracture

Age	Age 35-45 (n=58643)		Age46-55 (n=77054		Age56-65 (n=54972)		Total Male (n=190669)	
Fracture Type	Cases	Risk (%)	Cases	Risk (%)	Cases	Risk (%)	Total Cases	Total Risk (%)
Leg	504	17	521	14	355	13	1,380	14
Hip	77	3	133	3	98	4	308	3
Ankle	770	26	791	21	524	19	2,085	22
Spine	142	5	148	4	110	4	400	4
Wrist	933	32	922	24	631	23	2,486	26
Arm	620	21	645	17	397	14	1,662	17
Other	4,581	156	4,134	107	2,125	77	10,840	114

eResults 4: **Fracture Incidence by Age Group and Sex**

	Age 35-45			Age 46-55			Age 56-65		
Sex	Total Fractures (35-45)	Person Years (35-45)	Incidence (35-45)	Total Fractures (46-55)	Person Years (46-55)	Incidence (46-55)	Total Fractures (56-65)	Person Years (56-65)	Incidence (56-65)
Ankle									
Female	183	318,959	5.74	761	1,042,474	7.30	1,564	1,628,811	9.60
Male	226	268,760	8.41	467	832,178	5.61	635	1,330,018	4.77
Arm									
Female	123	319,175	3.85	591	1,043,809	5.66	1,418	1,631,113	8.69
Male	283	268,461	10.54	740	830,634	8.91	993	1,327,701	7.48
Hip									
Female	15	319,515	0.47	156	1,046,157	1.49	753	1,636,123	4.60
Male	47	269,372	1.74	168	834,890	2.01	434	1,333,395	3.25
MOF									
Female	358	318,531	11.24	1,699	1,038,658	16.36	4,816	1,612,238	29.87
Male	489	267,854	18.26	1,188	827,397	14.36	1,981	1,320,770	15.00
Other									
Female	271	318,651	8.50	970	1,040,940	9.32	1,675	1,628,053	10.29
Male	798	266,580	29.93	1,654	822,754	20.10	2,259	1,317,002	17.15
Spine									
Female	65	319,375	2.04	237	1,045,328	2.27	430	1,636,845	2.63
Male	96	269,184	3.57	298	833,985	3.57	518	1,332,384	3.89
Wrist									
Female	213	318,966	6.68	939	1,042,228	9.01	2,826	1,623,493	17.41
Male	196	268,822	7.29	384	832,702	4.61	540	1,331,045	4.06

eResults5: ***Number (Percentage) of Self-Reported Fractures from Fall - UKBB 2001-2006 by age and sex***

Sex	Age Group	Leg Fracture	Hip Fracture	Ankle Fracture	Spine Fracture	Wrist Fracture	Arm Fracture	Other Fracture
Female	35-45	146(53%)	16(40%)	581(75%)	33(30%)	615(71%)	337(71%)	1448(45%)
	46-55	364(64%)	97(70%)	1280(81%)	87(40%)	1716(81%)	893(79%)	2920(58%)
	56-65	365(70%)	120(68%)	1175(84%)	96(52%)	1888(85%)	761(81%)	2215(66%)
Male	35-45	153(30%)	25(33%)	381(50%)	45(32%)	413(44%)	261(42%)	1280(28%)
	46-55	227(44%)	56(42%)	456(58%)	36(24%)	498(54%)	328(51%)	1510(37%)
	56-65	188(53%)	50(51%)	347(66%)	31(28%)	407(65%)	254(64%)	928(44%)

References for Supplementary info

Bennett, D.A. (2001) ‘How can I deal with missing data in my study?’, *Australian and New Zealand Journal of Public Health*, 25(5), pp. 464–469.

Moreau, D. and Gamble, B. (2022) ‘Conducting a meta-analysis in the age of open science: Tools, tips, and practical recommendations’, *Psychological Methods*, 27(3), pp. 426–432. Available at: https://doi.org/10.1037/met0000351.

Morin, S.N., Lix, L.M. and Leslie, W.D. (2014) ‘The Importance of Previous Fracture Site on Osteoporosis Diagnosis and Incident Fractures in Women: PREVIOUS FRACTURE SITE AND INCIDENT FRACTURES’, *Journal of Bone and Mineral Research*, 29(7), pp. 1675–1680. Available at: https://doi.org/10.1002/jbmr.2204.

Rolls, C, (first), Clark, E and Munafo, M. R., last (2024) ‘Epidemiology of fractures in adults aged 35-60 in the UK biobank using self-reported fracture data and hospital episode statistics from 2006-2023.’ Available at: osf.io/q9p6s.

Sterne, J.A.C. *et al.* (2009) ‘Multiple imputation for missing data in epidemiological and clinical research: potential and pitfalls’, *BMJ*, 338, p. b2393. Available at: https://doi.org/10.1136/bmj.b2393.
